# Environment-Dependent Heterosis and Transgressive Gene Expression in Reciprocal Hybrids between the Channel Catfish *Ictalurus punctatus* and the Blue Catfish *Ictalurus furcatus*

**DOI:** 10.3390/biology11010117

**Published:** 2022-01-12

**Authors:** Haolong Wang, Timothy J. Bruce, Baofeng Su, Shangjia Li, Rex A. Dunham, Xu Wang

**Affiliations:** 1 Department of Pathobiology, College of Veterinary Medicine, Auburn University, Auburn, AL 36849, USA; hzw0088@auburn.edu; 2 Alabama Agricultural Experiment Station, Auburn, AL 36849, USA; tjb0089@auburn.edu (T.J.B.); BZS0014@auburn.edu (B.S.); szl0164@auburn.edu (S.L.); dunhara@auburn.edu (R.A.D.); 3 School of Fisheries, Aquaculture and Aquatic Sciences, Auburn University, Auburn, AL 36849, USA; 4 HudsonAlpha Institute for Biotechnology, Huntsville, AL 35806, USA

**Keywords:** heterosis, heterobeltiosis, environment, RNA-Seq, transcriptomics, transgressive genes, aquaculture

## Abstract

**Simple Summary:**

The hybrid catfish, generated by crossing female channel catfish and male blue catfish, has occupied the majority of the market share due to superior performance in growth rate, yield, and disease resistance in pond culture. However, we found that channel catfish have the best growth performance in tank units of smaller size, indicating that the heterosis is environment-dependent. To investigate the mechanisms of this intriguing phenomenon, hematological assays and transcriptome analysis were performed in the parental species and hybrid crosses. Lower levels of innate immunity activity, stress, as well as lowered blood glucose/lactate were found in channel catfish, which are associated with superiority in growth. Functional enrichment analysis revealed that genes involved in fatty acid metabolism/transport pathways are significantly upregulated in channel catfish. The results provide insights into the molecular mechanisms of heterosis and will inform the development of new strategies for genetic enhancement through hybrid breeding.

**Abstract:**

The hybrid between female channel catfish (*Ictalurus punctatus*) and male blue catfish (*Ictalurus furcatus*) is superior in feed conversion, disease resistance, carcass yield, and harvestability compared to both parental species. However, heterosis and heterobeltiosis only occur in pond culture, and channel catfish grow much faster than the other genetic types in small culture units. This environment-dependent heterosis is intriguing, but the underlying genetic mechanisms are not well understood. In this study, phenotypic characterization and transcriptomic analyses were performed in the channel catfish, blue catfish, and their reciprocal F_1_s reared in tanks. The results showed that the channel catfish is superior in growth-related morphometrics, presumably due to significantly lower innate immune function, as investigated by reduced lysozyme activity and alternative complement activity. RNA-seq analysis revealed that genes involved in fatty acid metabolism/transport are significantly upregulated in channel catfish compared to blue catfish and hybrids, which also contributes to the growth phenotype. Interestingly, hybrids have a 40–80% elevation in blood glucose than the parental species, which can be explained by a phenomenon called transgressive expression (overexpression/underexpression in F_1_s than the parental species). A total of 1140 transgressive genes were identified in F_1_ hybrids, indicating that 8.5% of the transcriptome displayed transgressive expression. Transgressive genes upregulated in F_1_s are enriched for glycan degradation function, directly related to the increase in blood glucose level. This study is the first to explore molecular mechanisms of environment-dependent heterosis/heterobeltiosis in a vertebrate species and sheds light on the regulation and evolution of heterosis vs. hybrid incompatibility.

## 1. Introduction

Interspecific hybrids are formed by crossing two distinct species. The hybrid offspring are sometimes superior in yield, size, growth rate, strength, fertility, or longevity traits than their parents. This phenomenon was first reported by Charles Darwin [1], and it is described by the following three terms depending on which trait it is referring to and how it is calculated: (1) hybrid vigor, when referring to fitness or reproductive traits leading to increased output of offspring [2]; (2) heterosis, the superiority of hybrids in production traits over the parental mean, which is measured by the average of the reciprocal F1s minus the parental mean divided by the parental mean; (3) heterobeltiosis, a special case of heterosis when the hybrids’ traits exceed the best performing parent [3], which is also known as Dunham’s practical heterosis [4,5]. In agricultural practices, many plant and animal breeds exhibit heterosis through breeding practices [6]. One of the most successful applications of selective breeding is the crossbred maize (*Zea mays*), with a higher grain yield [7]. In mammals, the benefits of heterosis are significant. For instance, post-weaning body weight gain in cattle increased by 1.49 kg in the hybrid of Continental (*Bos taurus*) × Zebu (*Bos indicus*) and by 14.68 kg in the British (*Bos taurus*) × Zebu (*Bos indicus*) hybrids [8]. For fertility traits, hybrid vigor was around 10–25% in crossbred dairy cattle [9]. Sheep survival was improved from 8.8% to 14.6% by crossbreeding among 14 different breeds [10]. In poultry, bodyweight at different development stages and egg production in F1 crossbred chicken were increased by 3.76–22.33% and 8.25%, respectively [11].

From an evolutionary perspective, the formation of new species occurs as populations diverge. Before speciation, there is a short period (heterosis phase) in which hybrid fitness is higher than that of the two parental species [12]. Thus, hybrid vigor may facilitate speciation by increasing adaptation to hostile environments, which has important implications in evolution and speciation. Variations in adaptive traits are heavily influenced by changes in gene expression [13]. Cumulative genetic variation and stabilizing selection of gene expression lead to coevolution, and thus molecular functions are preserved [14]. Crosses between species can disturb this coevolution and result in hybrid incompatibility (also called genetic incompatibility) [15]. In the hybrids, the expression level of most genes is close to the mid-parent value (additive effect) or near the level of one parent (dominance or partial dominance). Hybrid incompatibility is the manifestation of gene expression misregulation, resulting in expression levels higher (positive overdominance) or lower (negative overdominance) than both parental species [16]. Genetic loci with such expression patterns are called transgressive genes [17], which directly contribute to the superior phenotypes in heterosis or misregulation of gene expression in the hybrid breakdown or outbreeding depression [18].

The effect of gene expression regulation is often asymmetric in the two reciprocal F_1_ hybrid crosses. For example, gene regulatory evolution studies on frogs (crosses from *Xenopus laevis* and *X. muelleri*) [19], fish (reciprocal crosses among centrarchid species) [20], and bird (zebra finch subspecies) [21] showed asymmetric patterns of heterobeltiosis in one but incompatibility in the other reciprocal hybrids. The reduction in the fitness of hybrids is generally caused by Dobzhansky–Muller incompatibilities in the evolution field [22]. For example, the interaction between Lethal hybrid rescue (*Lhr*) and Hybrid male rescue (*Hmr*) in F_1_ hybrid males leads to lethality in the interspecific crosses between *Drosophila melanogaster* and *D. simulans* [23]. Interestingly, the genetic diversity between two parental species has a considerable effect on the performance of hybrid offspring. As the genetic distance increases, stronger heterosis effects may be observed in some cases [24]. However, when the two parental species are too distantly related, detrimental effects surpass heterosis, resulting in genetic incompatibility [25]. A further study shows the advantage of heterobeltiosis is partially countered by the disadvantage of genetic incompatibility [26].

Although the phenomenon of heterosis has been reported for more than a century, the underlying genetic mechanisms are still poorly understood. Three theories about heterosis have been proposed, which were widely discussed and debated: (1) Dominance hypothesis, the dominant allele suppresses the expression of the recessive allele according to Mendel’s theory [27]. The deleterious effects from recessive alleles carried by parental gametes were suppressed, and only the effects of beneficial dominant alleles were maintained; (2) Overdominance hypothesis, the expression of heterozygotes outperformed the highest parental expression values [28]; (3) Epistatic hypothesis, gene or allele interactions are existing and result in two conditions of epistasis: positive epistasis which causes the phenotype to be better than predicted, and negative epistasis in which performance is lower than expected. Positive epistatic interactions between non-allelic genes were thought to contribute to heterosis [29,30]. According to studies on gene transcription, expression differences between hybrid progeny and the parents, especially non-additive expression, contribute to heterosis [31]. However, existing theories of genetics basic do not give a unified explanation for both heterosis and hybrid incompatibility, which appear to be contradictory [32]. To understand the underlying genetic mechanisms, additional studies in different hybrid systems are needed.

Interspecific hybridization can mix genetic materials from two different species, which was proven to be an effective way to increase phenotypic variability and achieve genetic improvement in F_1_s [33]. In the breeding of aquatic species, interspecific hybridization, and crossbreeding within species have been applied as an effective means of genetic enhancement in a few cases. For example, crossbreeding has successfully improved the genetic stocks of common carp [34]. The sturgeon aquaculture industry was greatly promoted by the interspecific cross of Beluga (*Huso huso*) and sterlet (*Acipenser ruthenus*) because the hybridization reduced the sexual maturity time from 20 years to 6–9 years [35,36]. The growth rate of interspecific hybrids between the black drum (*Pogonias cromis*) and red drum (*Sciaenops ocellatus*) was significantly higher than both parents [37]. Hybrid progenies of the red abalone (female *Haliotis rufescens*) and the pink abalone (male *H. corrugate*) have significantly elevated growth and survival during conditions of thermal stress (22 °C) [38]. Hybrid striped bass was found to be superior in survival rate than the two parent species, white bass (*Morone chrysops*) and striped bass (*Morone saxatilis*). The hybrid bass has an intermediate growth rate (better than white bass) and thermal tolerance (better than stripe bass), and it is preferred for aquaculture [39]. Thus, aquaculture can greatly benefit from heterobeltiosis or overall greater performance in controlled hybridizations, allowing producers to use these enhanced attributes to increase production metrics and yields.

As the largest finfish aquaculture in the US [40], catfish accounts for 70% of total US freshwater production, and it is one of the most successful examples of the application of heterosis in aquaculture. Researchers evaluated 42 different interspecific ictalurid catfish hybrids by crossing two distinct species. Only the combination of channel catfish (*Ictalurus punctatus*) female × blue catfish (*Ictalurus furcatus*) male (C×B hybrid) has superior feed conversion efficiency [41,42], higher carcass yield [43], better tolerance to low oxygen [44], improved disease resistance [45], and enhanced harvestability [46] to market size. Collectively, these characteristics exhibiting heterobeltiosis enable a commercial production rate of 13,000 kg ha^−1^, which doubles the yield of traditional channel catfish farming [42,47,48]. Nowadays, the C×B hybrid catfish constitutes more than 50% of the total catfish harvest in the US [49].

In this study, the characteristics in C×B hybrid were found to be not heterotic in tank culture. Instead, the channel catfish parent was the superior genetic type in the aquarium environment. This finding suggested that the C×B heterobeltiosis was only observed in pond culture, which was an instance of environment-dependent heterobeltiosis/incompatibility. This phenomenon was previously reported in *Drosophila*, in which hybrid heterosis was higher in optimal density than in a crowded environment, and much higher at a lower than optimal temperature [50]. To understand the molecular basis of environment-dependent heterosis, growth-related morphometric traits were measured, including total length, standard length, body depth, body weight, head length, head depth, head width, and caudal depth, as well as physiological and immune parameters in channel catfish, blue catfish, and their reciprocal crosses under the tank culture. Liver transcriptome analyses were conducted to identify differentially expressed and transgressive genes, which provide insights into the molecular basis of environment-dependent heterosis.

## 2. Materials and Methods

### 2.1. Fish Maintenance and RNA-Seq Sample Collection

**Figure 1:**
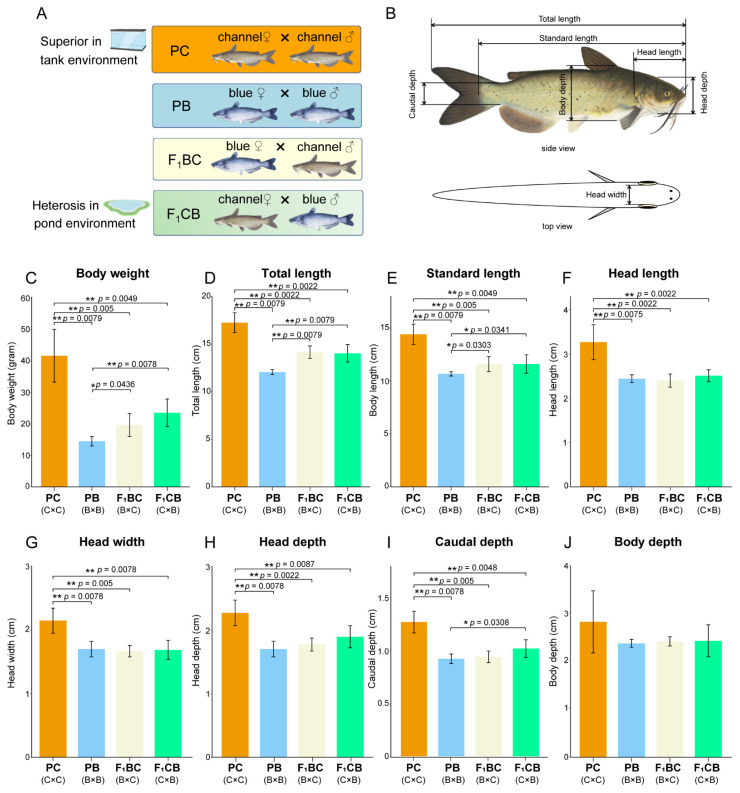
Morphometric measurements of channel catfish (C), *Ictalurus punctatus*, blue catfish (B), *I. furcatus,* and their reciprocal F1 hybrids raised in the tank environment. (**A**) Schematic illustration of four genetic cross types: channel catfish (C) parental cross (PC), blue catfish (B) parental cross (PB), blue catfish female × channel catfish male hybrids (F_1_BC), and channel catfish female × blue catfish male hybrids (F_1_CB) (**B**); Morphometric traits measured in this study: body weight (**C**); total length (**D**); body length (**E**); head length (**F**); head width (**G**); head depth (**H**) caudal depth (**I**); and body depth (**J**). Statistical significance was assessed by nonparametric Mann-Whitney U test (*, *p* < 0.05; **, *p* < 0.01;). The different colors representing the four genetic types were used consistently in this and subsequent figures.

All experimental animal protocols, including animal care and tissue sample collections, were approved by the Auburn University Institutional Animal Care and Use Committee (AU-IACUC). Blue catfish (PB), channel catfish (PC), B×C hybrid catfish (F_1_BC), and C×B hybrid catfish (F_1_CB) were reared at the Auburn University Fish Genetics Research Unit (Auburn, AL, USA; Figure 1A). The indoor unit has a recirculatory aquaculture system (RAS) equipped with mechanical and biological filters to clean and recycle rearing water to the fish culture tanks. Dissolved oxygen was maintained above 5 mg L^−1^, pH between 7.0 and 7.3, and water temperatures between 25 and 27 °C. One hundred fish from each group were maintained in separate 60 L rectangular tanks. At 12 months of age, two randomly selected fish from each genetic type were euthanized with buffered tricaine methanesulfonate (MS-222, Syndel Inc., Ferndale, WA, USA) for RNA-seq experiments. Liver tissues were immediately dissected, flash-frozen in liquid nitrogen, and stored in a − 80 °C freezer.

### 2.2. Morphometric Measurements

Six fish (fingerlings in July 2020) were randomly selected from each genetic type, and morphometric traits were measured at 10 months of age, including total length, standard length, body depth, body weight, head length, head depth, head width, and caudal depth (Figure 1B). Statistical significance among PB, PC, F_1_BC, and F_1_CB was assessed using the non-parametric Mann–Whitney U test. The significant *p*-value was shown by using asterisk rating system: *, *p* < 0.05; **, *p* < 0.01; ***, *p* < 0.001.

### 2.3. Biochemical and Immunological Assays

At 10 months of age, six fish for each genetic type (PB, PC, F_1_BC, and F_1_CB) were randomly selected. The total length of the fish ranged from 11.9 to 18.4 cm. Catfish were anesthetized with buffered MS-222 (100 mg L^−1^), and blood samples were collected from the caudal vasculature with BD U-100 syringes and transferred to lithium heparin-containing blood collection tubes (Becton Dickinson and Company, Franklin Lakes, NJ, USA). To obtain plasma, blood samples were immediately centrifuged at 1000× *g* for 10 min. Plasma glucose concentration was determined using Liquid Glucose (Oxidase) Reagent Set (Pointe Scientific Inc, Canton, MI, USA) using a 450 nm wavelength, with an input of 10 µL plasma per replicate. Lactate level was quantified by the Lactate (Liquid) Reagent Set (Pointe Scientific Inc, Canton, MI, USA) using a 595 nm wavelength and a sample input volume of 10 µL plasma per replicate. Lysozyme activity was determined based on the lysis of lysozyme-sensitive Gram-positive bacterium *Micrococcus lysodeikticus* (Sigma, St. Louis, MO, USA) by lysozyme present in the plasma according to Sankaran and Gurnani [51]. A total of 10 μL plasma was used as input, and 250 μL of bacterial cell suspension was added. The initial and final (after 30 min incubation at 37 °C) absorbances of the samples were measured at 450 nm. The rate of reduction in absorbance of samples was converted to lysozyme concentration (μg mL^−1^) using a standard curve. Alternative complement hemolytic activity (ACH50) was detected following a microplate protocol previously described by Welker [52]. The input of plasma was 25 µL per replicate, and the absorbance of the samples was measured at 405 nm.

### 2.4. Total RNA Extraction, RNA-Seq Library Preparation, and Sequencing

Two biological replicates were included for each of the four genetic types at 12 months of age (PB, PC, F_1_BC, and F_1_CB). RNA extraction was conducted using AllPrep DNA/RNA Mini Kit (Qiagen, Redwood City, CA, USA) following the manufacturer’s protocol. RNA concentrations were quantified using a NanoDrop OneC Microvolume Spectrophotometer (Thermo Scientific, Waltham, MA, USA). The RNA integrity was evaluated with the LabChip GX Touch HT (PerkinElmer, Hopkinton, MA, USA). The RNA library preparation was performed using NEBNext Poly(A) mRNA Magnetic Isolation Module and NEBNext Ultra II RNA Library Prep Kit for Illumina (New England BioLabs, Ipswich, MA, USA) with 1 µg of total RNA input. Purified mRNA samples were fragmented for 10 min at 94 °C. The first-strand cDNA synthesis, second-strand cDNA synthesis, end repair, and adaptor ligation were performed according to the manufacturer’s protocol. The library PCR amplification was performed with 16 cycles. The average size of the RNA libraries was approximately 350 bp (including the sequencing adapters). The RNA sequencing libraries were checked using the LabChip GX Touch HT (PerkinElmer, Hopkinton, MA, USA) and quantified using a Qubit 3.0 Fluorometer (Thermo Fisher Scientific, Waltham, MA, USA). The libraries were sequenced using a 2 × 150 Paired-End configuration in an Illumina NovoSeq 6000 lane at Novogene (Novogene Corporation Inc., Sacramento, CA, USA).

### 2.5. RNA-Seq Data Analysis and Identification of Differentially Expressed Genes among Channel Catfish, Blue Catfish, and Their Reciprocal F1 Hybrids

The quality of raw sequence data was checked by FastQC (version 0.11.5) [53]. Low-quality base and adapter sequences were trimmed by Trimmomatic (version 0.36) with default parameters [54]. Trimmed reads of 30 bp or longer were retained and mapped to the channel catfish reference genome [55] using TopHat (version 2.1.1) [56]. BedTools (version 2.29.0) [57] was used to quantify read counts that mapped to gene models. Differentially-expressed genes (DEGs) among four genetic types were identified using the edgeR package in R (version 3.6.3) [58]. The expression values of each gene were calculated as Reads Per Kilobase of transcript, per Million mapped reads (RPKM). Adjusted *p*-values were computed using the Benjamini and Hochberg method [59] with a threshold of 0.05. The thresholds for detecting significant DEGs were |log2FC (fold change)| > 1.5 and an adjusted *p*-value < 0.05.

### 2.6. Gene Ontology and Functional Enrichment Analysis for DEGs among PC, PB, and Reciprocal Hybrids F_1_BC and F_1_CB

For the DEGs from each pairwise comparison, Gene Ontology (GO) terms and KEGG (Kyoto Encyclopedia of Genes and Genomes) pathways enrichment analysis were performed by Metascape [60] with default parameters. The gene IDs were determined according to the homology to zebrafish annotations. GO analyses on biological processes, cellular components, and molecular functions were performed at an adjusted *p*-value cutoff of 0.01.

### 2.7. Identification and Functional Pathway Analysis of Transgressive Genes in Reciprocal F_1_ Hybrids

Transgressive genes were identified as genes with F_1_ hybrid expression levels at least 20% higher (or lower) than that in both parents (blue catfish and channel catfish). These transgressive genes were further classified into concordant and discordant transgressive genes based on the expression pattern in the reciprocal hybrids. Genes with higher (or lower) expression levels than the channel (PC) and blue catfish parents (PB) in both reciprocal hybrids (F_1_BC and F_1_CB) are defined as concordant transgressive genes, and the remaining genes are discordant. Among the discordant genes, the Discordant I category includes genes that are only transgressive in one hybrid cross but not the other. Discordant II genes show the opposite directions in the expression level in reciprocal F_1_ hybrids. These three subtypes of transgressive genes and transgressive genes in F_1_BC or F_1_CB hybrid catfish were subject to GO term and KEGG pathway enrichment analyses using Metascape. All analyses are carried out based on the knowledge base associated with the zebrafish at an adjusted *p*-value cutoff of 0.01.

### 2.8. Quantitative Reverse Transcription PCR Validation of DEGs and Transgressive Genes

A 400 ng aliquot of total RNA from each liver sample was reverse-transcribed using the LunaScript^®^ RT SuperMix Kit (New England BioLabs, Ipswich, MA, USA) with Oligo dT Primer in a 20 µL reaction, according to the manufacturer’s instructions. Six candidate genes were selected to be verified from DEGs and transgressive genes. Primer sequences were designed using the Oligo 7.0 software (Molecular Biology Insights Inc., Cascade, CO, USA). The primers were synthesized by Eurofins (Eurofins Genomics LLC., Louisville, KY, USA), and the amplification performance was checked by agarose gel electrophoresis. The qRT-PCR was performed in 96-well plates on a Bio-Rad C1000 Touch Thermal Cycler with CFX96 Real-Time PCR Detection Systems (Bio-Rad Laboratories, Hercules, CA, USA). The PCR reaction was performed in 20 µL systems using Luna^®^ Universal qPCR Master Mix (New England BioLabs, Ipswich, MA, USA). Each well contained 10 µL of Luna Universal qPCR Master Mix, 8 µL of nuclease-free water, 0.5 µL of each primer (10 µmol/L), and 1 µL of cDNA template. The reaction conditions were 95 °C for 60 s, followed by 40 cycles at 95 °C for 15 s and 60 °C for 30 s. After PCR amplification, a melting curve was generated by heating from 65 to 95 °C with 0.5 °C increments, 3 s dwell time, and a plate read at each temperature. All qRT-PCR assays were carried out with two technical replicates.

### 2.9. Statistical Analysis

For morphometric measurements, the one-way analysis of variance (ANOVA) was conducted to test for differences among four types of catfish. A Tukey post hoc test was carried out to compare morphometric measurements and hematological parameters (glucose, lysozyme, lactate, and ACH50) between genetic types. Mann–Whitney U test was used to compare morphometric data, hematological parameters level, and gene expression level measured by qRT-PCR for pairwise comparisons of two genetic types in the main figures. Statistical significances were determined at the *p* < 0.05 level.

## 3. Results

### 3.1. Environment-Dependent Heterobeltiosis—Channel Catfish Is Superior in Aquarium Culture

Channel catfish female × blue catfish male cross displayed a series of heterobeltiosis characteristics in pond culture [42]. To investigate whether the heterosis pattern holds in aquarium culture, we measured eight morphometric traits (Table S1 and Data S1) of 10-month old fish for each of the four genetic types (PB, PC, F_1_BC, and F_1_CB). The body weight of the channel catfish (41.3 g) was 2~3 fold higher than the other three genotypes (14.4 g, 19.5 g, and 23.3 g in PC, F_1_BC, and F_1_CB), indicating the channel catfish was superior in growth (*p* < 0.01; Figure 1C). Total length and standard length were also measured (Figure 1D,E), and channel catfish grew significantly faster in length than blue catfish and the reciprocal hybrids within 10 months of age in tank culture (*p* < 0.01). The head shape metrics (head length, head width, and head depth) and caudal depth showed the same pattern (*p* < 0.01; Figure 1F–I), which were known to be highly correlated. There was also a trend of wider body depth in channel catfish (2.83 ± 0.65 cm) than PC, F_1_BC, and F_1_CB, but the results did not achieve statistical significance (*p* > 0.05; Figure 1J).

When the body metrics were standardized by total length (TL), PB was significantly higher than F_1_BC (adjusted *p*-value < 0.05, Mann–Whitney U test; Figure S1) in all six body metrics (standard length, body depth, head length, head width, head depth, and body weight). PB also had a slightly elevated standard length/TL than all three other genetic types, and an increased head length/TL than F_1_CB (Figure S1). All other pairwise comparisons of normalized body shape parameters were not significant. As shown in the radar chart, the values of seven body metrics in channel catfish were higher than the three other genetic types (Figure S2). The results clearly demonstrated that heterosis in growth was not observed in C×B hybrid as expected in the pond environment, and channel catfish was the superior genomic configuration in tank culture.

### 3.2. Low Level of Innate Immunity and Complement Activities in Channel Catfish Raised in the Aquarium Environment

**Figure 2:**
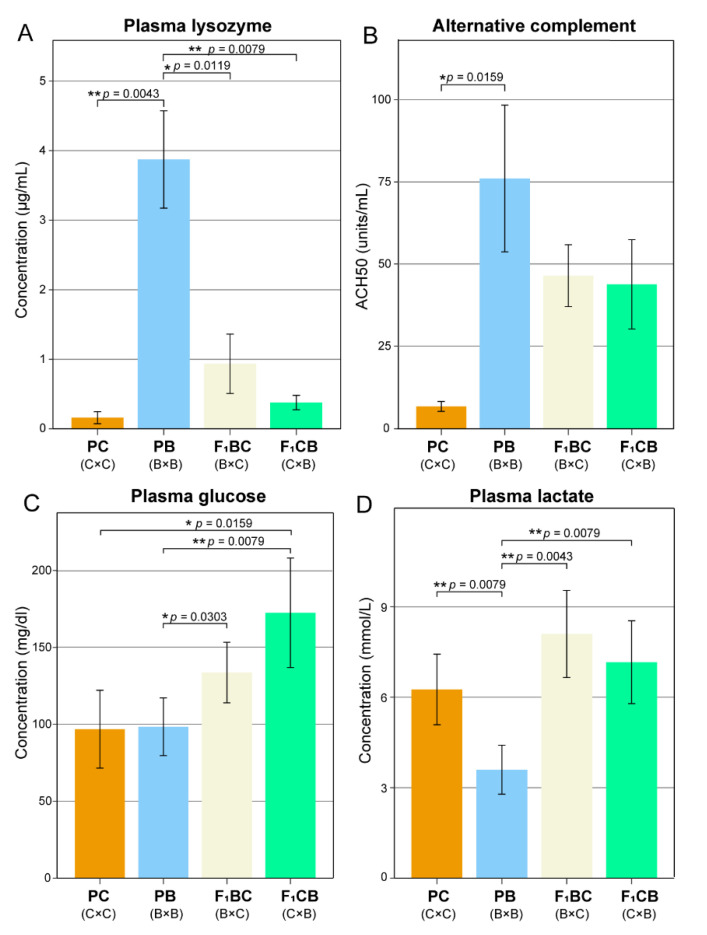
Plasma biochemical and immunological measurements in channel catfish (C), *Ictalurus punctatus*, blue catfish (B), *I. furcatus,* and their reciprocal F_1_ hybrids raised in the tank environment. Plasma lysozyme activity (**A**); alternative complement pathway hemolytic activity (**B**); plasma glucose level (**C**); and plasma lactate level (**D**) were measured in channel catfish parental cross (PC), blue catfish parental cross (PB), blue catfish female × channel catfish male hybrids (F_1_BC), and channel catfish female × blue catfish male hybrids (F_1_CB). Statistical significance was assessed by non-parametric Mann–Whitney U test (*, *p* < 0.05; **, *p* < 0.01).The different colors representing the four genetic types were used consistently in this and subsequent figures.

Lysozyme activity is a key index used to evaluate fish innate immune system activity [61]. Channel catfish plasma lysozyme activity was ~24-fold lower than blue catfish (*p* = 0.004), ~6-fold lower than the C×B hybrid (*p* = 0.008), and 2.4-fold lower than the B×C hybrid (*p* = 0.012; Figure 2A, Table S2, and Data S2). The complement system is an important component of the innate immune system, enhancing the ability to clear microbes and foreign cells [62], and the alternative pathway of this system is commonly measured. Blue catfish had the highest ACH50, 11-fold higher than channel catfish (*p* = 0.016; Figure 2B, Table S2, and Data S2). The reciprocal F_1_ hybrids were near the mid-parent value (Figure 2B, Table S2, and Data S2).

### 3.3. Transgressive Effects in Metabolism—F_1_ Hybrids Have Significantly Higher Blood Glucose Lactate Levels Than both Channel and Blue Catfish Parents

The blood glucose levels in the channel catfish and blue catfish were around 100 mg/dl (Figure 2C). The reciprocal F_1_ hybrids had significantly higher blood glucose levels than both parents: F_1_BC (133.6 mg/dl) was 38% higher than the parental species, and F_1_CB (172.5 mg/dl) was 78% higher (*p* < 0.05; Figure 2C and Table S2), indicating a transgressive effect in which the F_1_s had significant upregulation in blood glucose. Blood lactate was known to correlate with hyperactivity, stress level, and mortality in fish species. A similar transgressive effect was observed for the blood lactate level: both F_1_BC and F_1_CB were significantly higher than blue catfish (*p* < 0.01; Figure 2D and Table S2), and they were also higher than channel catfish, but it did not achieve statistical significance (Figure 2D).

### 3.4. Transcriptome Analysis in the Channel and Blue Catfish Parents and the Reciprocal F_1_ Hybrids Revealed >2000 Differentially Expressed Genes

**Figure 3:**
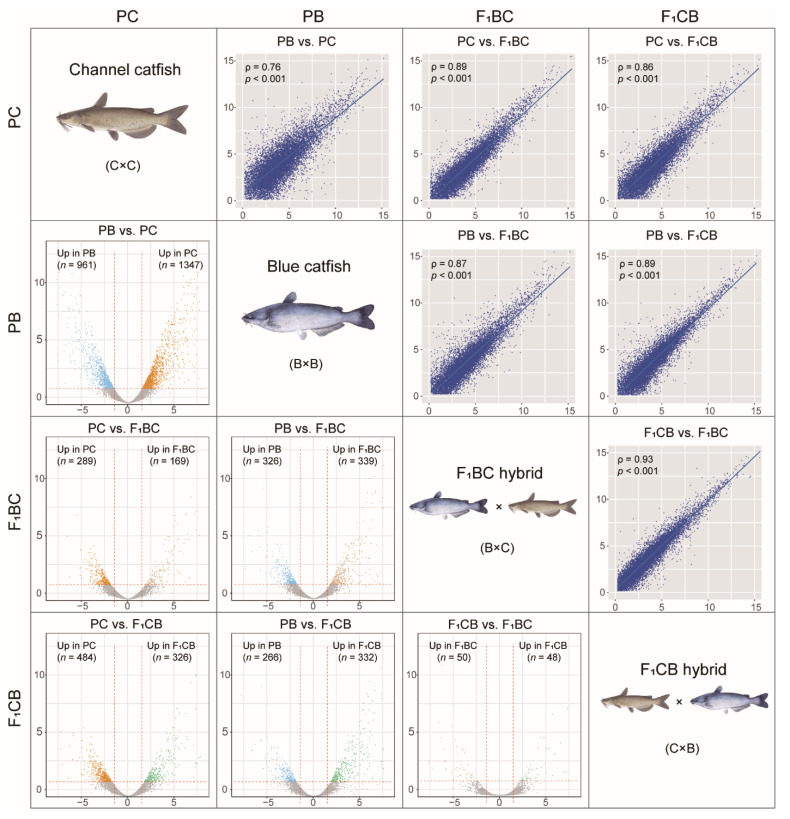
Transcriptome-wide gene expression correlation and differentially expressed genes in the liver among channel catfish (C), *Ictalurus punctatus*, blue catfish (B), *I. furcatus*, and their reciprocal F_1_ hybrids raised in the tank environment (***diagonal panels***). ***Bottom-left*** panels: volcano plots of six pairwise comparisons among the four genetic types from channel catfish parental cross (PC), blue catfish parental cross (PB), blue catfish female × channel catfish male hybrids (F_1_BC), and channel catfish female × blue catfish male hybrids (F_1_CB). Differentially expressed genes (DEGs) are highlighted (FDR < 0.05). The *x*-axis stands for log_2_ fold changes, and the *y*-axis represents −log_10_(*p*-value). The vertical lines indicate |log_2_FoldChange| = 1.5. ***Upper-right*** panels: scatterplots of the log_2_ (RKPM) values for six pairwise comparisons among the four genetic types. Spearman’s rank correlation coefficient *ρ* and the corresponding *p*-values are labeled.

Liver RNA-seq analyses were performed on channel catfish (PC), blue catfish (PB), C×B hybrid catfish (F_1_CB), and B×C hybrid catfish (F_1_BC) (Table S3). A total of 13,420 expressed genes were identified with Reads Per Kilobase of transcript, per Million mapped reads (RPKM) value greater than 1.0 in at least one genetic type. Pairwise differential gene expression analysis was conducted to identify the differentially expressed genes (DEGs) between two genetic types (Figure 3 and Table S4). There were more DEGs between the two parental species (*n* = 2308; FDR < 0.05 and |log2FoldChange| > 1.5) than the parent-hybrid comparisons (458~810 DEGs), which was consistent with the fact that PC and PB had the lowest transcriptome-wide gene expression correlation (spearman correlation coefficient *ρ* = 0.76; Figure 3). The reciprocal hybrids F_1_CB and F_1_BC only had 98 DEGs with the highest expression correlation (*ρ* = 0.93; Figure 3). Since the F_1_ hybrids had identical nuclear genome configurations, and they were expected to display similar gene expression profiles. The results confirmed that this was the case. The evolutionary divergence and genetic distance between the channel and blue catfish resulted in gene expression changes of >2000 genes (Data S3), which account for 17.2% of expressed genes in the liver transcriptome.

### 3.5. Fatty Acid Metabolism and Transport Genes Were Significantly Upregulated in Channel Catfish Compared to Blue Catfish and Hybrids

**Figure 4:**
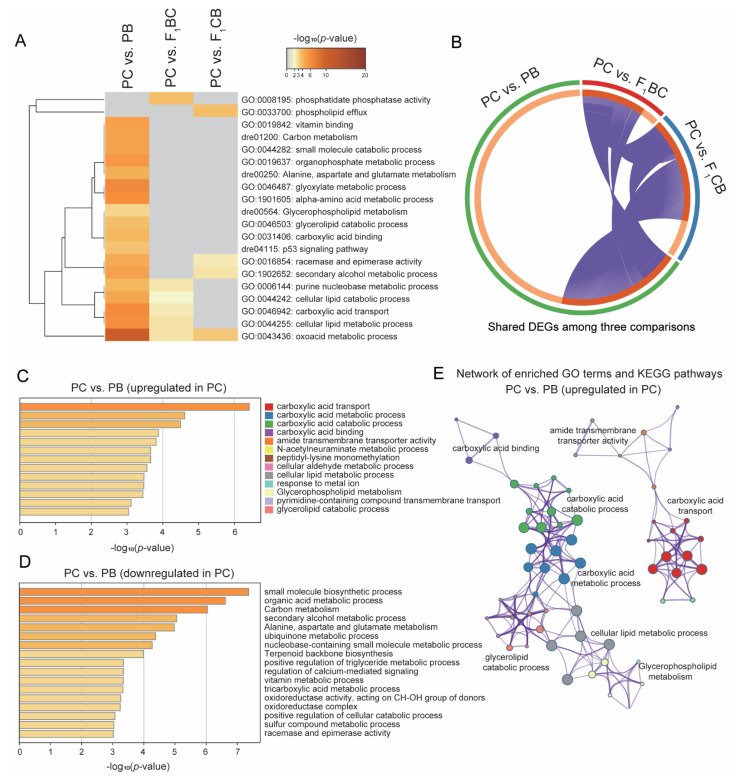
Pathway enrichment analysis of liver Differentially Expressed Genes (DEGs) between channel catfish *Ictalurus punctatus* parental cross (PC) and three other genetic types (PB: blue catfish *I. furcatus* parental cross, blue catfish female × channel catfish male hybrids (F_1_BC), and channel catfish female × blue catfish male hybrids (F_1_CB). (**A**) Hierarchical clustering of significant gene ontology terms shared in at least two of the three comparisons (PC vs. PB, PC vs. F_1_BC, and PC vs. F_1_CB); (**B**) A circular plot of shared DEGs in the three comparisons; (**C**,**D**) Enriched functional categories for upregulated genes in PC compared to PB (**C**) and downregulated genes in PC compared to PB (**D**); Enrichment scores measured by −log_10_(*p*-value) were shown on the *x*-axis; (**E**) A plot of enriched term network for upregulated genes in PC. GO terms were represented by the same color dots as in (**C**), and the interconnectivity was represented by the edges.

Gene ontology (GO) and Kyoto Encyclopedia of Genes and Genomes (KEGG) pathways enrichment analyses were performed to identify the enriched functional pathways among DEGs. Among the identified terms, a third of the PC-PB significant GO categories were also significant in PC-F_1_ comparisons (Figure 4A,B), including cellular lipid metabolic processes (GO:0044255), cellular lipid catabolic processes (GO:0044242), secondary alcohol metabolic process (GO:1902652), and oxoacid metabolic process (GO:0043436; Figure 4A). Specific non-overlapping terms enriched only in PC-F_1_ comparisons included phospholipid efflux (GO: 0033700) in the PC-F_1_CB comparison, as well as phosphatidate phosphatase activity (GO: 0008195) in the PC-F_1_BC comparison (Figure 4A). These findings suggested that the lipid metabolism pathway activities were altered in PC.

Since channel catfish is superior in aquarium growth than PB and hybrids, we focused our analysis on the significantly upregulated and downregulated genes in PC compared to PB (*p* < 0.001; Figure 4C,D). Interestingly, the PC upregulated genes were enriched for four carboxylic acid pathways (metabolic, catabolic, transport, and binding; Figure 4C) and three lipid metabolism pathways (cellular lipid metabolic process, glycerophospholipid metabolism, and glycerolipid catabolic process; Figure 4C). The network analyses revealed that the fatty acids and lipid metabolism genes were interconnected (Figure 4E), which was separated from the transporter-related terms (Figure 4E), suggesting these two broader functional categories were significantly overrepresented in PC upregulated genes. With regard to the genes that were significantly highly expressed in PB, the top three enriched functional terms are small-molecule biosynthesis, organic acid metabolic process, and carbon metabolism (Figure 4D), which are in sharp contrast to PC upregulated genes.

### 3.6. One Thousand Genes Displayed Transgressive Expression Patterns in the Liver of F_1_ Hybrid Catfish

**Figure 5:**
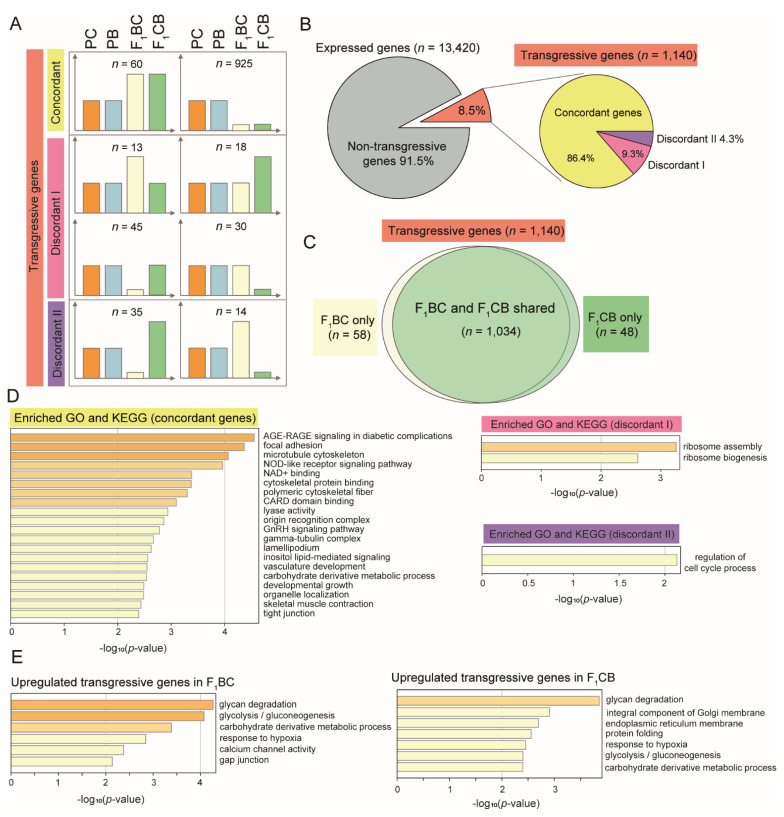
Identification and functional enrichment analysis of liver transgressive genes in the reciprocal hybrids of channel catfish, *Ictalurus punctatus*, and blue catfish, *I. furcatus*. (**A**) Definition of different classes of transgressive genes based on the gene expression levels in channel catfish parental cross (PC), blue catfish parental cross (PB), blue catfish female × channel catfish male hybrids (F_1_BC), and channel catfish female × blue catfish male hybrids (F_1_CB). The *y*-axis represents relative gene expression levels. The gene counts were labeled for each class; (**B**) Piechart of transgressive gene distributions in hybrid catfish; (**C**) Venn diagram of transgressive genes in F_1_BC and F_1_CB hybrids; (**D**) Enriched functional categories for concordant and discordant transgressive genes; (**E**) Enriched functional categories for upregulated transgressive genes in F_1_BC and F_1_CB hybrids. Enrichment scores measured by −log_10_(*p*-value) were shown on the *x*-axis.

**Figure 6:**
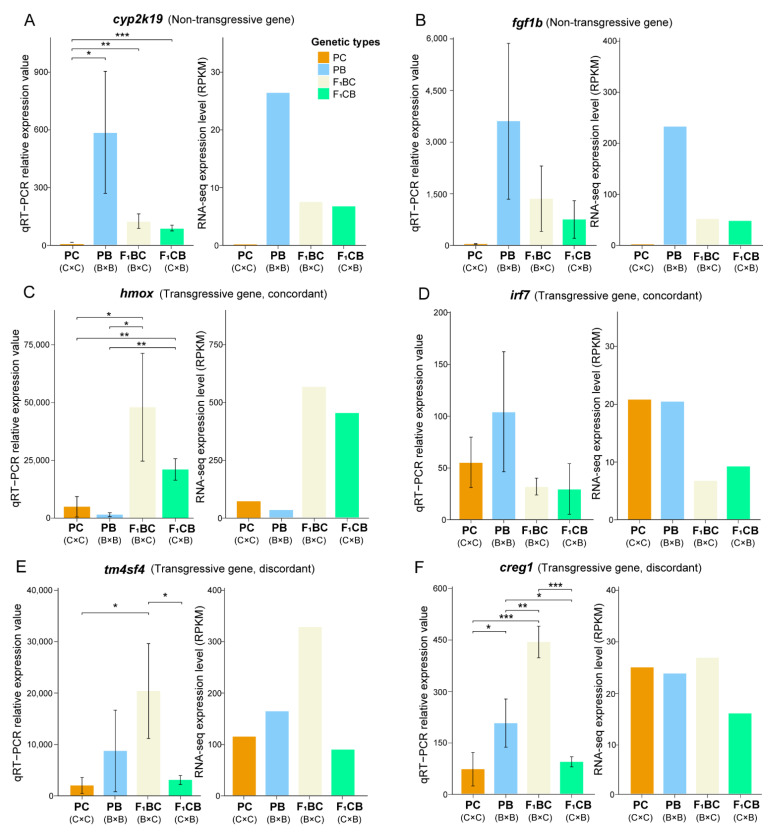
Quantitative reverse transcription PCR validation of differentially expressed genes and transgressive genes. PC: channel catfish *Ictalurus punctatus* parental cross; PB: blue catfish I. furcatus parental cross; F_1_BC: blue catfish female × channel catfish male hybrids; F_1_CB: channel catfish female × blue catfish male hybrids. Barplots of qRT-PCR relative quantification and RNA-seq RPKM values (Reads Per Kilobase of transcript per Million mapped reads) for non-transgressive genes *cyp2k1* (**A**) and *fgf1* (**B**), concordant transgressive genes *hmox* (**C**) and *irf7* (**D**), and discordant transgressive genes *tm4sf4* (**E**) and *creg1* (**F**). Mann–Whitney U test was used to assess the statistical significance (*, *p* < 0.05; **, *p* < 0.01; ***, *p* < 0.001).

To elucidate the molecular basis of heterobeltiosis vs. hybrid incompatibility, a group of genes called transgressive genes were investigated, which were defined as genes that show higher or lower expression levels in both parents (see Materials and Methods). According to the transgressive pattern in reciprocal hybrids, the transgressive genes were further classified as (1) concordant: transgressive genes in both F_1_ hybrids with the same direction, which are higher than both parents (upregulated concordant genes) or lower than both parents (downregulated concordant genes); (2) discordant I: genes that are transgressive in only one reciprocal F_1_, but not the other (Figure 5A); (3) discordant II: genes that are transgressive in both F_1_s, but the expression directions are opposite (Figure 5A). A total of 1140 transgressive genes were identified in F_1_ hybrids, which count for 8.5% of all expressed genes in the liver transcriptome (Figure 5B and Data S4). Over 90% of the transgressive genes were shared in F_1_CB and F_1_BC (Figure 5C), suggesting that the cross direction-dependent transgressive effect (discordant I genes) only occurred in less than 10% of transgressive genes (Figure 5A,B). Even fewer genes displayed the opposite pattern in gene expression changes compared to the parental species, and these discordant II genes (*n* = 49) accounted for 4.3% of all transgressive genes (Figure 5B). To validate the transgressive genes detected in RNA-seq experiments, qRT-PCR experiments for gene expression quantifications for six genes were performed (Table S5), and all of them were confirmed, including two non-transgressive genes *cyp2k19* and *fgf1b* (Figure 6A,B), two concordant genes *hmox* and *irf7* (Figure 6C,D), as well as two discordance genes *tm4sf4* and *creg1* (Figure 6E,F).

### 3.7. Concordant Transgressive Genes in Hybrid Catfish Were Enriched for Cytoskeleton Functions, Stress, and Immune-Related Pathways

Six cytoskeleton and extracellular matrix-related terms were enriched in concordant transgressive genes, including microtubule cytoskeleton (GO: 0015630), cytoskeletal protein binding (GO: 0008092), polymeric cytoskeletal fiber (GO: 0099513), focal adhesion (dre04510), gamma–tubulin complex (GO: 0000930), and lamellipodium (GO: 0030027). Oxidative stress and immune functions were also significantly overrepresented in concordant genes. The most significantly enriched term was AGE-RAGE signaling (dre04933; *p* < 0.0001; Figure 5D), which plays an important role in inflammation in diabetes. The Nod-like receptors (dre04521; *p* < 0.001; Figure 5D) are master regulators of inflammation and defense, which activate innate and adaptive immunity by recognizing pathogen patterns. CARD domain binding genes (GO: 0050700; *p* < 0.001; Figure 5D) are often associated with inflammation and apoptosis. NAD+ binding (GO: 0070403; *p* < 0.001; Figure 5D) function is involved in cellular energy metabolism. Discordant I transgressive genes were enriched for ribosome function (*p* < 0.001; Figure 5D), the term regulation of cell cycle process (GO: 0010564) was overrepresented in discordance II genes (*p* < 0.01; Figure 5D).

### 3.8. Overrepresentation of Glycan Degradation Function among Upregulated Transgressive Genes Provided a Potential Mechanism for the Blood Glucose Elevation in F_1_ Hybrids

For the concordant transgressive genes (*n* = 985), the majority of them (94%) were downregulated in both F_1_ hybrids, whereas only 60 concordant genes were upregulated compared to the channel catfish and blue catfish parents (Figure 5A). These overexpressed genes may explain the transgressive phenotypes observed in the F_1_ hybrids (Figure 2). GO and KEGG analysis of upregulated transgressive genes in F_1_BC and F_1_CB were performed. Interestingly, the top enriched term was the same, which was glycan degradation (dre00511; *p* < 0.0001; Figure 5E). Glycan breakdown in the liver will result in an elevation in blood glucose level in both hybrids F_1_BC and F_1_CB compared to the channel catfish and blue catfish parents, which was what we observed (Figure 2C).

## 4. Discussion

### 4.1. The Phenomenon of Environment-Dependent Heterosis in Hybrid Catfish

The degree of heterosis was known to be affected by the environment and genotype-environment interactions [63]. Environment-influenced heterosis has been reported in cattle, in which the advantage of milk and protein yields was suppressed in a high HLI (summer heat load index) environment [64]. Examples of environment-influenced heterosis also can be found in aquatic species. Crossbred offspring from two silver perch (*Bidyanus bidyanus*) strains from Murray River and Cataract Dam had the best performance in growth under pond environment, whereas heterosis decreased when reared in cages and tanks [65]. In these situations, the degree of heterosis was affected by the rearing environment, but heterosis did not disappear entirely. We define this phenomenon as environment-influenced heterosis.

Environment-dependent heterosis was first discovered in *Drosophila* in 1987. Crosses among five geographically diverse *D. melanogaster* inbred lines identified significant heterosis in fecundity [50]. When the flies were maintained at a higher than optimal density, heterosis was still observed for all hybrid crosses with a slightly lesser degree, indicating that crowdedness can affect hybrid vigor, but it does not abolish heterosis [50]. However, when the flies were reared in the lower temperature (17 °C rather than 24 °C), heterosis was only present in two hybrid crosses, and all other hybrid line pairs had lower fecundity than the inbred crosses [50], suggesting heterosis disappeared for most hybrid crosses under low-temperature environment. The hybridization of the channel catfish and blue catfish is a vertebrate example of environment-dependent heterosis. The hybrid cross grew 30–121% faster than the channel catfish in pond environments with different densities, whereas the channel catfish’s mean body weight was 49.8% higher than hybrid fish when they were grown in cages [66]. Both environment-influenced and environment-dependent heterosis had been observed in hybrid catfish [66]. In this study, we discovered that the channel catfish was superior in body weight (Figure 1C) and all other morphometric traits (Figure S2) than blue catfish and the reciprocal hybrids, at 10 months of age in tank culture, although channel catfish did not differ from the reciprocal hybrids in standardized shape parameters (Figure S1). The reciprocal hybrids between the channel and blue catfish may serve as an excellent system to investigate environment-dependent heterosis and their molecular mechanisms.

### 4.2. The Biological Robustness and Prevalence of Transgressive Genes in Channel-Blue Catfish Hybrid System

Interspecific hybrids can display heterosis, but they could also suffer from hybrid necrosis [67] or hybrid incompatibility [68]. If the two parental genomes were too distantly related, DNA sequence and gene expression divergence would result in misregulation of protein-coding gene expression in F_1_s. Uneven chromosome numbers can also cause the hybrid breakdown. As a classic example of heterosis in mammals, a mule is an offspring of a female horse (2*n* = 64) and a male donkey (2*n* = 62). Although mules outperform both parents in many aspects, including strength, stamina, temper, and longevity, mules are infertile because the odd number of chromosomes will affect proper segregation during meiosis. Hybridization can also lead to the aberrant activation of transposable elements (TEs), which was known as “genome shock” discovered in maize by Barbara McClintock [69]. Genes with transgressive expression patterns may explain the heterosis vs. hybrid incompatibility. After merging the two parental genomes, the F_1_ gene expression level is expected to be near the mid-parental value (additive effect), or close to either parent (dominant or incomplete dominant effects), or within the parental range (both effects). In contrast, transgressive genes have higher (or lower) expression levels compared to both parents, which can explain the superior phenotypes or hybrid misregulation in F_1_s. In our study, over 90% of expressed genes were non-transgressive genes, and transgressive genes only accounted for 8%, which is consistent with the level of divergence between the channel catfish and blue catfish (13–15 SNPs per Kb) estimated from EST data [70]. The channel-blue hybrids are fully viable with strain-dependent variable fertility [71], suggesting major biological functions and metabolic/developmental pathways can tolerate transgressive genes in some crosses, exhibiting robustness at the organism level.

### 4.3. The Superiority in Tank Growth May Be Associated with Low Immune Activity and Stress Levels in Channel Catfish

In the pond culture, heterobeltiosis in growth, disease resistance, and harvestability was only observed in C×B hybrids [42]. In the tank environment, channel catfish is the fast-growing genetic type compared to all other three genomic configurations. Based on previous literature on the density-influenced heterosis in pond vs. cage culture in other fish species, the degree of crowding and/or accompanying stress levels was believed to play important roles in the variation in heterosis. However, density-dependent stress alone cannot explain the fact that hybrid catfish did show heterobeltiosis in tanks of larger sizes (>1 cubic meter), even if the density was extremely high. Therefore, our current results only apply to the smaller aquarium environment and cannot be generalized to larger tanks.

As important defense mechanisms in fish’s innate immune system [72], lysozyme and complement activity (including the alternative pathway) are widely used to evaluate the immunity and ability of the fish to clear pathogens [61,73]. In channel catfish, lysozyme activity was reported to correlate with blood bacteria concentrations after exposure to pathogenic *Edwardsiella ictaluri* [74]. Additionally, it has been previously reported that alternative complement activity can vary across fish species, and as a whole, catfish were found to have relatively low ACH50 values in comparison to barramundi (*Lates calcarifer*) and rabbitfish (*Siganus rivulatus*) [75]. Thus, innate immune parameters need to be compared within closely related fish species or strains.

In this research, lysozyme and alternative complement levels in channel catfish were more than 10-fold lower than blue catfish and significantly lower than the hybrids (Figure 2A,B), suggesting a dramatic decrease in innate immune activity in channel catfish in the absence of pathogenic infections under aquarium environment. This was consistent with previous findings using in vivo pathogen challenge experiments, in which channel catfish were found to be least resistant to bacterial pathogens overall compared to the blue catfish and F_1_CB (F_1_BC not tested). For the three major infectious diseases in catfish production, blue catfish was almost completely resistant to *Edwardsiella ictaluri,* the pathogen for Enteric Septicemia of Catfish (ESC), with 0.7–10.5% mortality [76,77]. F_1_CB had a 26% mortality under ESC [77], whereas the mortality for channel catfish was up to 72.3% [76] (resistance to ESC: PB >> F_1_CB > PC). For *Aeromonas* spp. infections, blue catfish (32% mortality) [78] were reported to be more resistant than F_1_CB [79], with channel catfish (90% mortality in [80] and 78% mortality in [81]) as the least resistant genetic type (resistance to Aeromonas disease: PB > F_1_CB >> PC). For columnaris disease, the hybrid F_1_CB was observed to be much more resistant (32% mortality) to *Flavobacterium columnare* than channel catfish (74% mortality) and blue catfish (87% mortality) [45] (resistance to columnaris disease: F_1_CB >> PC > PB). Similarly, Zhang et al. (2020) also demonstrated increased lysozyme levels in hybrid yellow catfish (female yellow catfish *Pelteobagrus fulvidraco ×* male darkbarbel catfish *P. vachelli*) compared to all-male yellow catfish [82]. Since maintaining a highly-activated immune system is both energetically and nutritionally expensive, which will inhibit growth and development, the lowered innate immunity activity discovered in the channel catfish may explain the superior phenotype in weight gain under aquarium culture in small tanks.

Elevated plasma glucose and lactate levels were observed in hybrid catfish in this study. Increased glucose levels in rainbow trout were reported to be attributed to environmental changes, such as season [83], density stocking [84], as well as nutritional changes or stressors [85]. Interestingly, a recent study in channel catfish evaluated the role of gastric peptides on glucose levels and discovered complex regulation of glucose levels [86]. Plasma lactate is also an important stress-related parameter and has been shown to increase in channel catfish when confined or deprived of oxygen [87]. With respect to stress management, stressed fish typically have higher plasma lactate levels, which could be the result of hyperactivity [64,88]. Metabolic activities, such as lipolysis and glycolysis, provide the energy required to meet the demands of the stress response, potentially resulting in a negative impact on growth. In summary, the lower plasma glucose and lactate levels may also contribute to the fast-growing phenotype of channel catfish in tank culture.

### 4.4. Fatty Acid and Lipid Metabolism Are Enriched in Channel Catfish

Among the upregulated genes in the channel catfish compared to blue catfish, the top four significant gene ontology terms were all related to carboxylic acid (*p* < 0.001; Figure 4C). Three lipid metabolism terms were also significant (*p* < 0.001; Figure 4C). Hydroxy-carboxylic acids (HCAs) are intermediates in animal energy metabolism, and HCA receptors play an important role in homeostasis by regulating energy metabolism, lipolysis, inflammation, and immunity [89]. HCAs were understudied in fish species, but HCA metabolic process was reported to be the most significant GO term between non-alcoholic fatty liver patients and controls [90], and research has shown that activated hydroxy-carboxylic acids (HCAs) inhibit adipocyte lipolysis [91,92], suggesting its relevance to lipid metabolism and storage. For many fish species, fatty acid oxidation is the primary source of energy. The enriched HCA and lipid metabolism-related terms in channel catfish upregulated genes may mediate the difference in immune function and growth phenotypes.

### 4.5. High Blood Glucose in Hybrids Is Likely to Be due to Glycan Degradation in the Liver

The liver is a crucial organ for maintaining glucose homeostasis. The blood glucose levels were found to be significantly higher in both F_1_s than the channel catfish and blue catfish (Figure 2C), and they also exceeded the normal range. Hyperglycemia is common in teleost species [93], but this transgressive effect may result in increased glucose utilization and reduced weight gain in F_1_s. To explore the molecular basis, transgressive genes that were upregulated in F_1_BC and F_1_CB were investigated. Interestingly, glycan degradation was identified as the top enriched pathway for both hybrids (Figure 5E). The glycolysis pathway was also overrepresented in the upregulated transgressive genes. These findings suggested that rapid glycan degradation and glucose utilization may explain the higher blood glucose level and reduced weight gain in the F_1_ hybrids in the small tank/aquarium environment.

### 4.6. Toward a Better Understanding of the Environment-Dependent Heterobeltiosis in Hybrid Catfish

The environment-dependent heterobeltiosis is an intriguing phenomenon. It is the first reported case in any vertebrate species, and it is of great interest in both evolutionary biology of heterosis and agriculture practice to enhance the catfish genetic stock. However, this is a complicated problem, and many aspects of it warrant further research. First of all, environmental variations can affect the status of heterosis. We have shown that in small tanks, channel catfish was the superior genetic type for growth and development. However, from the previous literature discussed in 4.3, this may not be the case under pathogenic infections, under which blue catfish and the C×B hybrids have better survival rates overall. Second, a “tank size” effect exists. In tanks larger than one cubic meter, heterobeltiosis was observed just as in the pond culture, independent of the fish density. Third, there might be an age, size, and density-dependent effect as well, since channel catfish grow the fastest during year 1 in low-density ponds, but hybrids grow faster than channel catfish in year 1 in high-density ponds. In contrast, this density-related relationship does not occur during year 1 growth in aquaria. To further complicate the situation, these factors interact with the genetic and epigenetic backgrounds of the four types, as well as neurological differences in complex social behavior, level of stress, physical activities, hormonal changes, innate immune attributes, feed intake and frequency, and flighting or tank hierarchy among individuals. Further studies are needed to disentangle these complex factors to elucidate the underlying mechanisms of this fascinating phenomenon.

## 5. Conclusions

Heterosis and heterobeltiosis are the genetic basis for production enhancement using interspecific hybrid breeding techniques. Hybrid catfish are superior in a number of production and disease-resistant traits, and they grow much faster than both channel catfish and blue catfish parents. Interestingly, this heterobeltiosis only occurs in pond culture, and channel catfish are superior in growth in smaller culturing units, such as tanks and aquaria. This research investigated this intriguing environment-dependent heterosis, and identified three potential mechanisms of this phenomenon: (1) significantly lower lysozyme activity and alternative complement activity discovered in channel catfish may reduce the energy cost of immune function to promote growth; (2) fatty acid metabolism/transport pathways were enriched in channel catfish upregulated genes, which may explain the faster growth of channel catfish than the other three genetic types; (3) F_1_ hybrids had elevated blood glucose levels compared to channel catfish, which may result from liver glycan degradation. Collectively, these gene expression and physiological differences contributed to the lack of heterosis in the tank culture environment. For the first time, this study provided insights into the regulation of environment-dependent heterosis in a vertebrate model. Further studies are needed to determine when and how the heterobeltiosis happened after the tank-pond environment transition.

## Data Availability

The raw RNA-seq data is available at NCBI GEO (Gene Expression Omnibus) databases under the accession number GSE186603.

## Supplementary Materials

The following supporting information can be downloaded at: https://www.mdpi.com/article/10.3390/biology11010117/s1. Figure S1: Morphometric measurements of channel catfish (C), *Ictalurus punctatus*, blue catfish (B), *I. furcatus*, and their reciprocal F1 hybrids raised in the tank environment, standardized by total length (TL); Figure S2: Radar plot of seven body metrics in channel catfish (C), *Ictalurus punctatus*, blue catfish (B), *I. furcatus*, and their reciprocal F1 hybrids raise in the tank environment; Table S1: Comparison of morphometric measurements for blue catfish, channel catfish, and their reciprocal hybrids in the tank environment; Table S2: Biochemical and immunological assays in blue catfish, channel catfish, and their reciprocal hybrids cultured in the tank environment; Table S3: Liver sample RNA sequencing yield, quality control, and alignment statistics; Table S4: Numbers of significantly differentially expressed genes (DEGs) in pairwise comparisons among four genetic types (PB, PC, F_1_BC, and F_1_CB); Table S5: List of primer sequences for quantitative reverse transcription PCR validation; Data S1: Raw measurements of morphometric characteristics for 10-month blue catfish, channel catfish, and their reciprocal hybrids cultured in the tank environment; Data S2: Raw biochemical and immunological measurements in blue catfish, channel catfish, and their reciprocal hybrids cultured in the tank environment; Data S3: List of differentially expressed genes from pairwise comparisons among PB, PC, F_1_BC, and F_1_CB; Data S4: List of transgressive genes in blue-channel catfish hybrids. Supplemental Data files are available at github.com/XuWangLab/2021_catfish_hybrid_heterosis_sppData (last accessed on 5 January 2022).
