# Supplementary material for: Environment-Dependent Heterosis and Transgressive Gene Expression in Reciprocal Hybrids between the Channel Catfish Ictalurus punctatus and the Blue Catfish Ictalurus furcatus
Source: Biology (Basel). 2022 Jan 12;11(1):117. doi: 10.3390/biology11010117 (PMC8772901; doi:10.3390/biology11010117)

Table S1. Comparison of morphometric measurements for blue catfish, channel catfish, and their reciprocal hybrids in the tank environment.

	Channel	Blue	B×C hybrid	C×B hybrid	F-value	Heterosis		Heterobeltiosis	
						B×C	C×B	B×C	C×B
**Total length (cm)**	17.25±1.03[c]	12.06±0.25[a]	14.15±0.66[b]	14.03±0.92[b]	40.88**	-3.45	-4.26	-17.97	-18.67
**Standard length (cm)**	14.40±0.96[b]	10.66±0.21[a]	11.58±0.71[a]	11.58±0.87[a]	25.78**	-7.58	-7.58	-19.58	-19.58
**Head length (cm)**	3.27±0.39[b]	2.44±0.09[a]	2.40±0.15[a]	2.51±0.14[a]	18.77**	-15.94	-12.08	-26.61	-23.24
**Head width (cm)**	2.15±0.20[b]	1.70±0.12[a]	1.67±0.09[a]	1.68±0.15[a]	14.77**	-13.25	-12.73	-22.33	-21.86
**Head depth (cm)**	2.27±0.20[b]	1.70±0.12[a]	1.77±0.10[a]	1.89±0.17[a]	15.02**	-10.83	-4.79	-22.03	-16.74
**Body depth (cm)**	2.83±0.65	2.38±0.08	2.43±0.10	2.43±0.33	1.79	-6.72	-6.72	-14.13	-14.13
**Caudal depth (cm)**	1.26±0.10[b]	0.92±0.04[a]	0.94±0.05[a]	1.02±0.08[a]	25.59**	-13.76	-6.42	-25.4	-19.05
**Weight (g)**	41.33±8.31[c]	14.40±1.52[a]	19.50±3.62[ab]	23.33±4.37[b]	29.01**	-30.02	-16.27	-52.82	-43.55

Significance of one-way ANOVA: *: $P<0.05$; **: $P<0.01$. Values in the same column having the same superscripts are not significantly different (Tukey's test, $P > 0.05$). Channel: channel catfish; Blue: blue catfish; B×C hybrid: female blue catfish × male channel catfish; C×B hybrid: female channel catfish × male blue catfish.

Heterosis= $\frac{\bar{F}1 - MPV}{MPV} \times 100$

Heterobeltiosis= $\frac{\bar{F}1 - BPV}{BPV} \times 100$

Where: $\bar{F}_1$ average of F1 hybrid; BPV: Better Parent Value; MPV: Mid Parent Value (P1+P2)/2.

Table S2. Biochemical and immunological assays in blue catfish, channel catfish, and their reciprocal hybrids cultured in the tank environment.

	Blue catfish	Channel catfish	C×B hybrid	B×C hybrid
Lactate (mmol/L)	3.599 ± 0.811[a]	6.263 ± 1.173[b]	7.168 ± 1.375[b]	8.104 ± 1.444[b]
Glucose (mg/dl)	98.385 ± 18.741[a]	96.788 ± 25.282[a]	172.462 ± 35.578[b]	133.615 ± 19.666[a]
Lysozyme (µg/mL)	3.872 ± 0.7[a]	0.160 ± 0.086[b]	0.378 ± 0.102[b]	0.935 ± 0.427[b]
ACH50 (units/mL)	76.179 ± 22.325[ac]	6.820± 1.496[b]	43.979 ± 13.584[c]	46.596 ± 9.410[c]

Note: values in the same row having the same superscripts are not significantly different (Tukey's test, $P > 0.05$). B×C hybrid: female blue catfish × male channel catfish; C×B hybrid: female channel catfish × male blue catfish.

Table S3. Liver sample RNA sequencing yield, quality control, and alignment statistics.

Sample ID	Sample name	Body weight at collection	Raw reads	Filtered reads	Mapping rate to channel genome	Mapping rate to blue genome
CxC_C58_indiv1	Channel catfish liver	47.6 g	74,645,394	72,939,430	79.6%	37.4%
CxC_C58_indiv2	Channel catfish liver	52.5 g	79,057,946	76,881,484	79.3%	29.7%
BxB_C57_indiv1	Blue catfish liver	20.2 g	89,934,258	87,650,476	37.3%	84.1%
BxB_C57_indiv2	Blue catfish liver	18.6 g	67,639,738	66,215,758	42.0%	81.9%
BxC_C66_indiv1	B×C hybrid liver	29.0 g	92,917,484	90,536,414	57.6%	68.1%
BxC_C66_indiv2	B×C hybrid liver	32.2 g	91,926,868	90,204,272	65.2%	71.2%
CxB_C56_indiv1	C×B hybrid liver	23.5 g	100,242,864	97,806,146	66.9%	55.6%
CxB_C56_indiv2	C×B hybrid liver	26.8 g	93,576,928	90,995,214	50.6%	60.4%

Table S4. Numbers of significantly differentially expressed genes (DEGs) in pairwise comparisons among four genetic backgrounds (PB, PC, F_1BC, and F_1CB.).

	Number of DEGs	Upregulated	Downregulated
PC vs. PB	2,307	1,346	961
PB vs. F_1BC	665	326	339
PB vs. F_1CB	598	266	332
PC vs. F_1BC	458	289	169
PC vs. F_1CB	810	484	326
F_1BC vs. F_1CB	98	50	48

Table S5. List of primer sequences for quantitative reverse transcription PCR validation.

Gene name	5'- 3' primer sequence	PCR product size (bp)
Cyp2k19-F	GGCCAAGTACCCTCACATACAGG	154
Cyp2k19-R	ACTCATGGGTACTATATTTGCCAG	
Fgf1b-F	ATCATCAGAGGTCACGAGGC	166
Fgf1b-R	CGTACCAGACCCCGTGCTC	
Hmox-F	GTCGCACCAATTTACTTCCCT	234
Hmox-R	GGTAATTCGGCCCAACACC	
Irf7-F	TATGATTTGCCAAACCACATGC	170
Irf7-R	ACAGTCATGTTATTCGGAAGG	
Creg1-F	CAAGCACAAAGGTTACGACCC	210
Creg1-R	CCTCCGAAGTAGTCCAACACC	
Tm4sf4-F	AGCTGGTTATTCTTTCATCGT	217
Tm4sf4-R	CAGAGCCATTTGAACCATGC	

Figure S1. Morphometric measurements of channel catfish (C), *Ictalurus punctatus*, blue catfish (B), *I. furcatus*, and their reciprocal F1 hybrids raised in the tank environment, standardized by total length (TL).

Morphometric traits normalized by total length for the four genetic cross types: channel catfish (C) parental cross (PC), blue catfish (B) parental cross (PB), blue catfish female × channel catfish male hybrids (F_1BC), and channel catfish female × blue catfish male hybrids (F_1CB). (**A**), body length; (**B**), body depth; (**C**), head length; (**D**), head width; (**E**), head depth; (**F**), caudal depth. Statistical significance was assessed by the nonparametric Mann-Whitney U test. (*, adjusted *P*-value < 0.05).

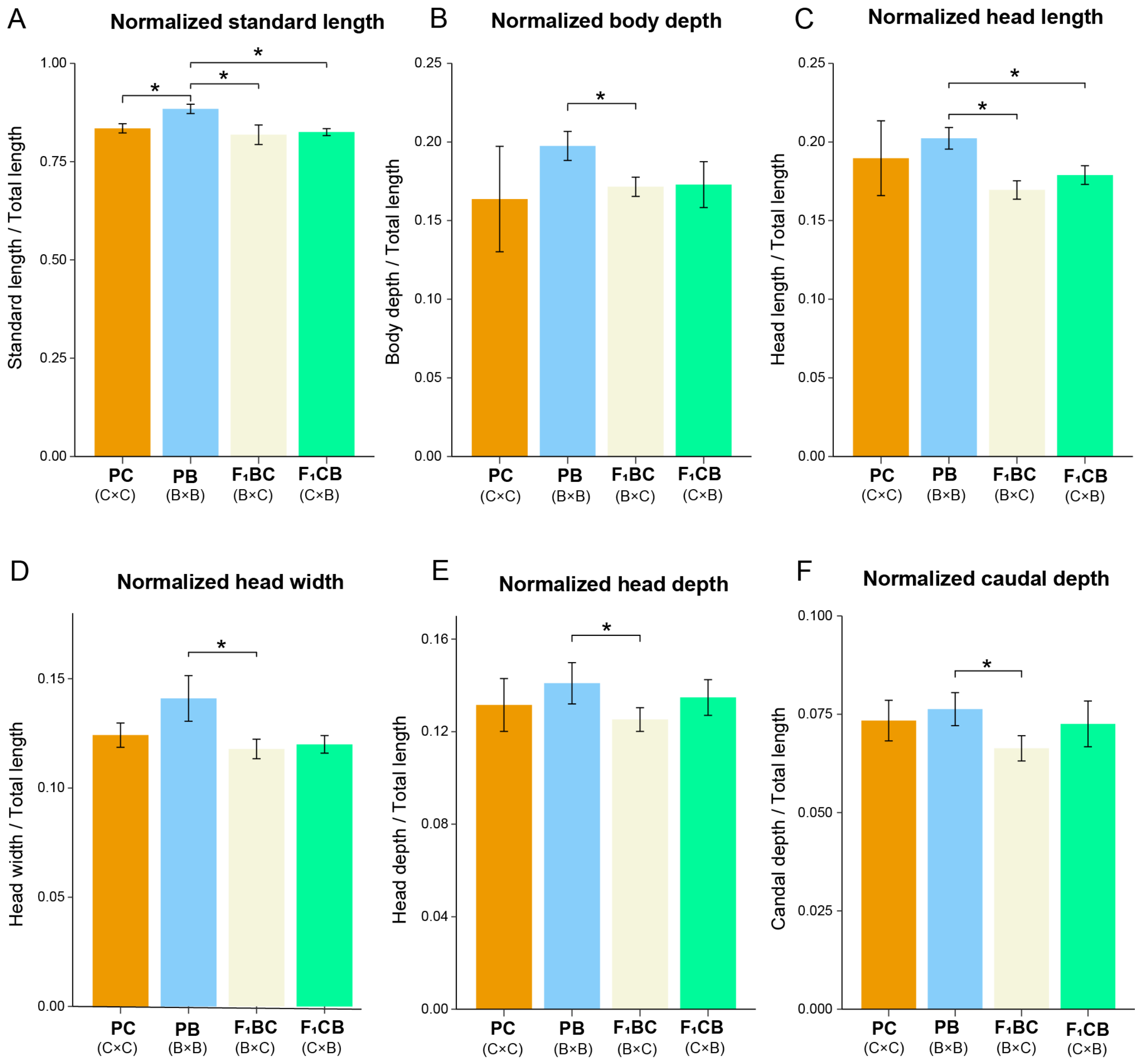

Figure S2. Radar plot of seven body metrics in channel catfish (C), *Ictalurus punctatus*, blue catfish (B), *I. furcatus*, and their reciprocal F_1 hybrids raise in the tank environment.

Radar chart for seven body measuremements (total length, body length, head length, head width, head depth, caudal depth) in channel catfish (C) parental cross (PC), blue catfish (B) parental cross (PB), blue catfish female × channel catfish male hybrids (F_1BC), and channel catfish female × blue catfish male hybrids (F_1CB). The channel measurement area is shaded in orange.

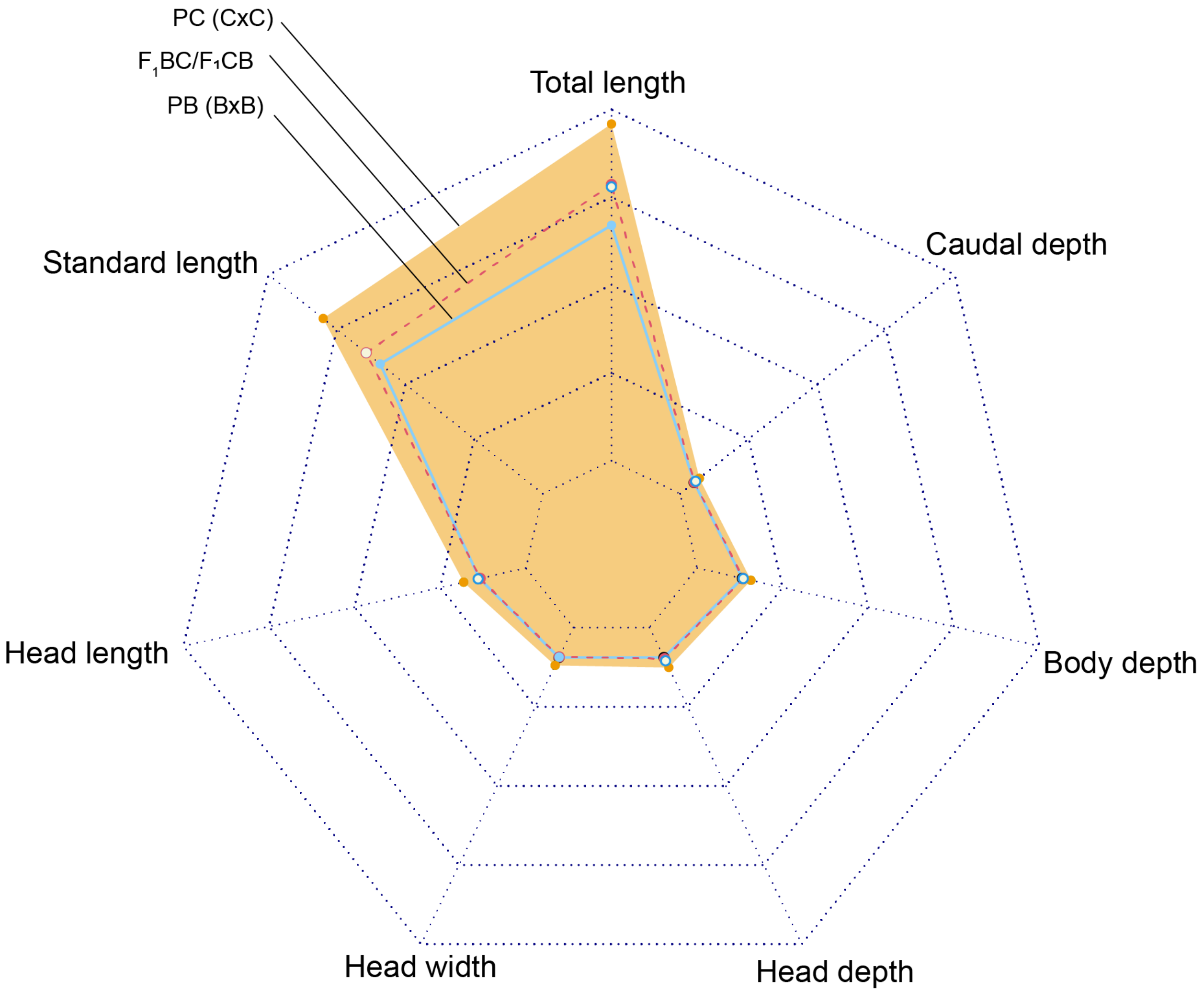

Supplement: Supplementary file 1 [file biology-11-00117-s001.zip › biology-1511795-supplementary.pdf]
